# Prognostic and clinicopathological role of geriatric nutritional risk index in patients with diffuse large B-cell lymphoma: A meta-analysis

**DOI:** 10.3389/fonc.2023.1169749

**Published:** 2023-03-30

**Authors:** Dan Cao, Zongxin Zhang

**Affiliations:** ^1^ Department of Hematology, Huzhou Central Hospital, Affiliated Central Hospital of Huzhou University, Huzhou, Zhejiang, China; ^2^ Clinical Laboratory, Huzhou Central Hospital, Affiliated Central Hospital of Huzhou University, Huzhou, Zhejiang, China

**Keywords:** geriatric nutritional risk index, meta-analysis, diffuse large B-cell lymphoma, prognosis, clinical practice

## Abstract

**Background:**

Previous studies have explored the relationship between the geriatric nutritional risk index (GNRI) and survival outcomes of diffuse large B-cell lymphoma (DLBCL) cases, but the results were inconsistent. Consequently, the present meta-analysis was conducted to investigate how GNRI affects DLBCL and its function in terms of prognosis.

**Methods:**

The Web of Science, PubMed, Embase, and Cochrane Library databases were thoroughly searched until January 18, 2023. We calculated combined hazard ratios (HRs) and 95% confidence intervals (CIs) to estimate the relationship between the GNRI and survival outcomes of patients with DLBCL.

**Results:**

This meta-analysis included seven articles involving 2,353 cases. A lower level of GNRI predicted dismal overall survival (HR=1.40, 95% CI=1.25–1.56, p<0.001) and inferior progression-free survival (HR=1.46, 95% CI=1.19-1.80, p<0.001) of DLBCL patients. Moreover, a low GNRI was significantly related to Eastern Cooperative Oncology Group Performance Status ≥2 (odds ratio [OR]=4.55, 95% CI=2.75–7.54, p<0.001), Ann Arbor stage III–IV (OR=2.91, 95% CI=2.38–3.57, p<0.001), B symptoms (OR=3.51, 95% CI=2.34–5.29, p<0.001), and extranodal disease (OR=2.90, 95% CI=2.32–3.63, p<0.001).

**Conclusion:**

A lower GNRI level predicted poorer short- and long-term prognosis in patients with DLBCL. A low GNRI was correlated with clinical factors of disease progression in DLBCL patients.

## Introduction

Among non-Hodgkin lymphoid (NHL) malignancies, diffuse large B-cell lymphoma (DLBCL) accounts for the highest proportion (30–40% of NHL cases) (1). Approximately 60% of the DLBCL cases can be treated using standard therapeutic regimens (such as rituximab, cyclophosphamide, doxorubicin, vincristine, and prednisone) (2). However, 45–50% of the cases relapse or become refractory after a complete response (3). The prognosis for patients experiencing relapse is poor because 80% of them ultimately die from DLBCL, even after treatment with subsequent regimens (4). The poor survival outcomes of DLBCL patients are partially due to the lack of effective prognostic markers. Therefore, identifying novel and readily available biomarkers is important for the prognosis of DLBCL.

Growing evidence has shown that nutritional status and immune responses play essential roles in tumor initiation, development, and metastasis (5, 6). Many parameters derived from laboratory examinations have drawn considerable attention because of their prognostic value. Recently, numerous studies reported the relationship between a series of serum-based parameters and the prognosis of DLBCL (7–10). These indexes include the lymphocyte-to-monocyte ratio (7), neutrophil-to-lymphocyte ratio (NLR) (8), C-reactive protein (9), and platelet-to-lymphocyte ratio (PLR) (10). The geriatric nutritional risk index (GNRI) is a nutritional marker that includes patient’s body weight (BW), height, and serum albumin content. It is calculated by the formula: GNRI = 1.487 × serum albumin (g/L) + 41.7 × present/optimal BW (kg) (11). In clinical settings, the GNRI is used as a simple nutrition evaluation approach, and a low GNRI indicates poor nutritional status of patients (12, 13). In recent years, numerous studies have analyzed the role of GNRI in predicting the prognosis of DLBCL cases (14–20), but their findings remain controversial. For example, in some studies, low GNRI significantly predicted poor survival in DLBCL patients (14, 16, 19). However, other researchers have reported that the GNRI is not related to DLBCL survival (15). Previous studies that adopted different cut-off values of the GNRI could also contribute to the conflicting results. Therefore, we searched recent literatures and carried out a meta-analysis to identify whether the GNRI predicted DLBCL prognosis accurately.

## Materials and methods

### Study guideline

This study was performed following the Preferred Reporting Items for Systematic Reviews and Meta-Analyses guidelines (21).

### Literature search

Studies were identified in the Web of Science, PubMed, Embase, and Cochrane Library databases. The search strategies were as follows: (geriatric nutritional risk index or GNRI) and (diffuse large B-cell lymphoma or DLBCL or lymphoma). Detailed search strategies for each database are provided in **Supplementary File 1**. Retrieval timeline was from inception until January 18, 2023. Only publications published in the English language were considered. Relevant documents in references of the identified studies were also searched.

### Eligibility criteria

Studies conforming to the following criteria were included (1): cases with a pathological diagnosis of DLBCL; (2) GNRI determined prior to anticancer therapy; (3) studies mentioning the function of GNRI in predicting prognosis, such as overall survival (OS), progression-free survival (PFS), recurrence-free survival (RFS), and cancer-specific survival (CSS); (4) studies with available hazard ratios (HRs) together with associated 95% confidence intervals (CIs) regarding patient outcomes; (5) studies with a threshold to classify high/low GNRI; and (6) articles written in the English language. Studies conforming to the following standards were excluded: (1) reviews, case reports, meeting abstracts, letters, and correspondences; (2) articles that included overlapping patients; and (3) animal studies.

### Data collection and quality evaluation

Two independent reviewers (D.C. and Z.Z.) were responsible for data collection from qualified articles. Any disagreement between the reviewers was resolved through mutual negotiation until a consensus was reached. The following data were collected: name of first author, country, publication year, sample size, age, sex, study duration, follow-up, threshold GNRI, threshold measurement approach, study center, survival analysis, survival endpoints, treatment, and HRs with 95% CIs. The methodological quality of the eligible articles was evaluated using the Newcastle–Ottawa scale (NOS) (22). Articles with NOS scores of ≥6 were regarded as high-quality articles.

### Statistical analysis

Combined HRs and 95% CIs were determined to estimate the relationship between the GNRI and survival outcomes in the DLBCL cases. Heterogeneity among the enrolled articles was analyzed using I^2^ statistics and the Cochran’s Q test. An I^2^ statistics of ≥50% and/or p<0.10 on the Cochran Q test indicated obvious heterogeneity, and the random-effects model was used; otherwise, the fixed-effects model was adopted. Diverse factor-stratified subgroup analyses were carried out to detect sources of heterogeneity. Correlations of GNRI with clinicopathological features in DLBCL were explored by combining odds ratios (ORs) and associated 95% CIs. The Begg’s test was used for publication bias, while an asymmetry assessment was performed using a funnel plot. All statistical analyses were carried out using Stata software (version 12.0; Stata Corporation, College Station, TX, USA). Statistical significance was set at p<0.05 (two-sided), which represented statistical significance.

### Ethnics statement

The need for ethical approval was waived from this work, and no informed consent was obtained because no patient information was involved.

## Results

### Study selection process

**Figure 1:**
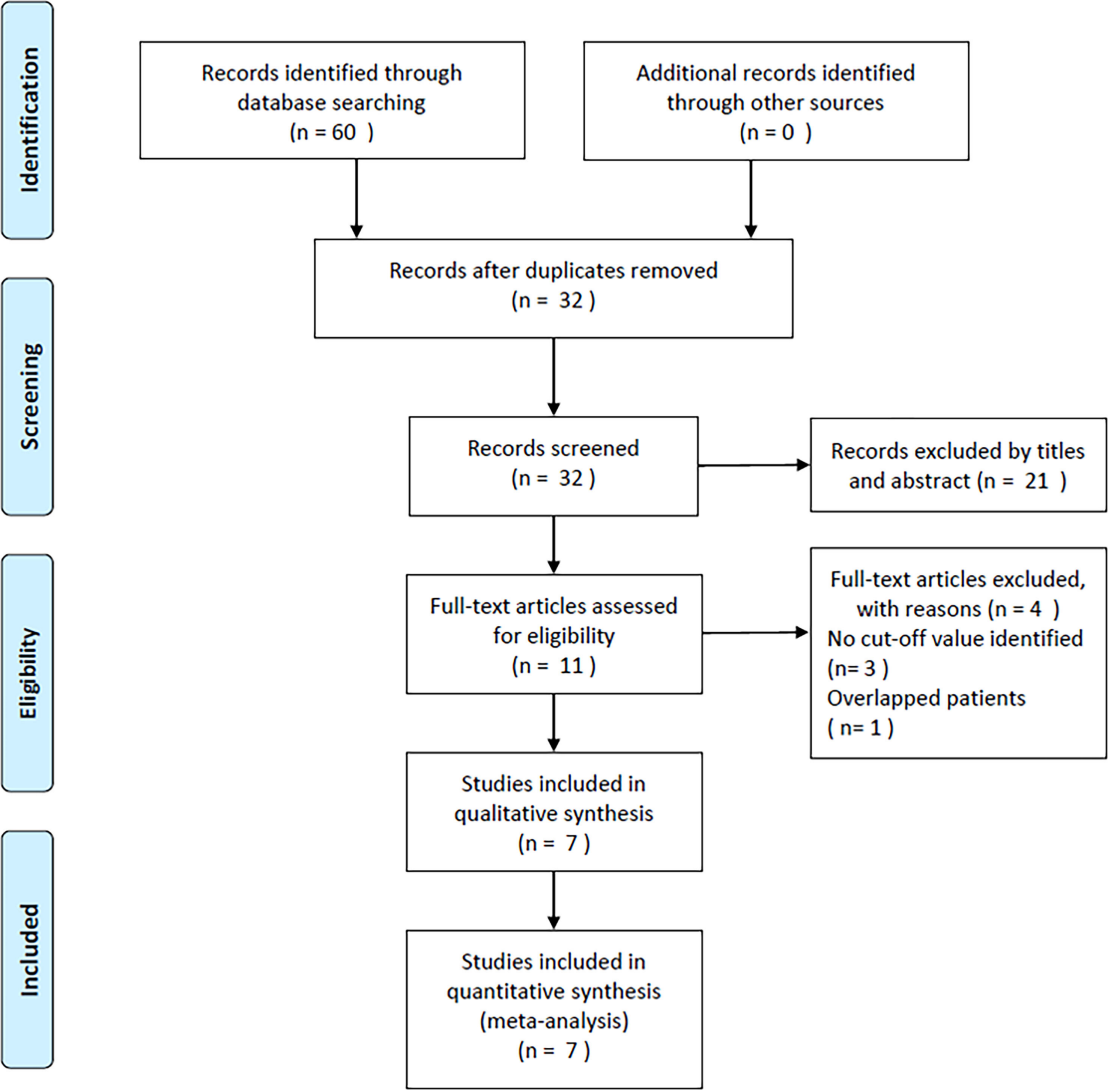
PRISMA flow diagram of the study selection process.

As shown in **Figure 1**, the original study selection detected 60 studies, and after removal of the duplicates, 32 records remained. Subsequently, titles and abstracts were scanned, and 21 articles were discarded because of their irrelevance. By reading the full texts of 11 articles, 4 articles were then eliminated due to no cut-off value of GNRI (n=3) and inclusion of overlapping patients (n=1). Finally, seven articles, involving 2,353 cases (14–20), were included in the present study (**Figure 1**; **Table 1**).

**Table 1:** Baseline characteristics of included studies in this meta-analysis.

Study	Year	Country/ region	Sample size	Age (years) Median(range)	Gender (M/F)	Study period	Ann Arbor stage	Treatment	Follow-up (month) Median(range)	Cut-off value	Cut-off determination	Study center	Survival endpoints	Survival analysis	NOS score
Kanemasa, Y.	2018	Japan	476	68(27-97)	266/210	2004-2017	I-IV	R-CHOP or R-CHP-COP	45	96.8	ROC curve	Single center	OS, PFS	Multivariate	8
Li, Z.	2018	China	267	59	156/111	2010-2016	I-IV	R-CHOP	1-72	98	Literature	Single center	OS	Multivariate	7
Matsukawa, T.	2020	Japan	615	69(20-97)	337/278	2008-2018	I-IV	R-CHOP	1-60	95.7	ROC curve	Multicenter	OS	Multivariate	7
Chuang, T. M.	2021	Taiwan	205	75(65-96)	107/98	2010-2019	I-IV	R-CHOP	1-140	92.5	ROC curve	Single center	OS, PFS	Multivariate	9
Lee, S.	2021	Japan	451	78(65-96)	223/228	2007-2017	I-IV	R-CHOP or R-CHP-COP	22.3(1-140.3)	92	ROC curve	Multicenter	OS	Multivariate	6
Yan, D.	2021	China	133	71(60-91)	67/66	2014-2018	I-IV	R-CHOP	35.2	106.26	ROC curve	Single center	OS	Univariate	7
Atas, U.	2022	Turkey	206	58.5	112/94	2008-2020	I-IV	R-CHOP	27.5(1-164)	104.24	ROC curve	Single center	OS	Multivariate	7

M, male; F, female; ROC, receiver operating characteristic; OS, overall survival; PFS, progression-free survival; NOS, Newcastle-Ottawa Scale; R-CHOP, rituximab with cyclophosphamide, doxorubicin, vincristine and prednisone; R-THP-COP, rituximab with cyclophosphamide, tetrahydropyranyl adriamycin, vincristine, prednisolone.

### Qualified article characteristics

**Table 1** shows the basic characteristics of the qualified articles. All the eligible articles were published between 2018–2022 (14–20). Three studies were conducted in Japan (14, 16, 18), two in China (15, 19), and one each in Taiwan (17) and Turkey (20). The sample size was 133–615 (median, 267). All the included studies had a retrospective design and enrolled DLBCL patients with Ann Arbor stage I–IV (14–20). The threshold GNRI was 92–106.26 (median, 96.8). Six studies analyzed thresholds with receiver operating characteristic (ROC) curves (14, 16–20), while one study selected cut-off values according to the literature (15). Five studies were carried out in a single center (14, 15, 17, 19, 20), and two were multicenter trials (16, 18). All seven articles mentioned the role of GNRI in predicting OS (14–20), and two studies reported an association between the GNRI and PFS (14, 17) in DLBCL. Six articles mentioned the HRs and 95% CIs through multivariate regression (14–18, 20), and one study adopted a univariate analysis (19). The NOS scores were 6–9 (median, 7), indicating high quality.

### Prognostic value of GNRI for OS and PFS

**Figure 2:**
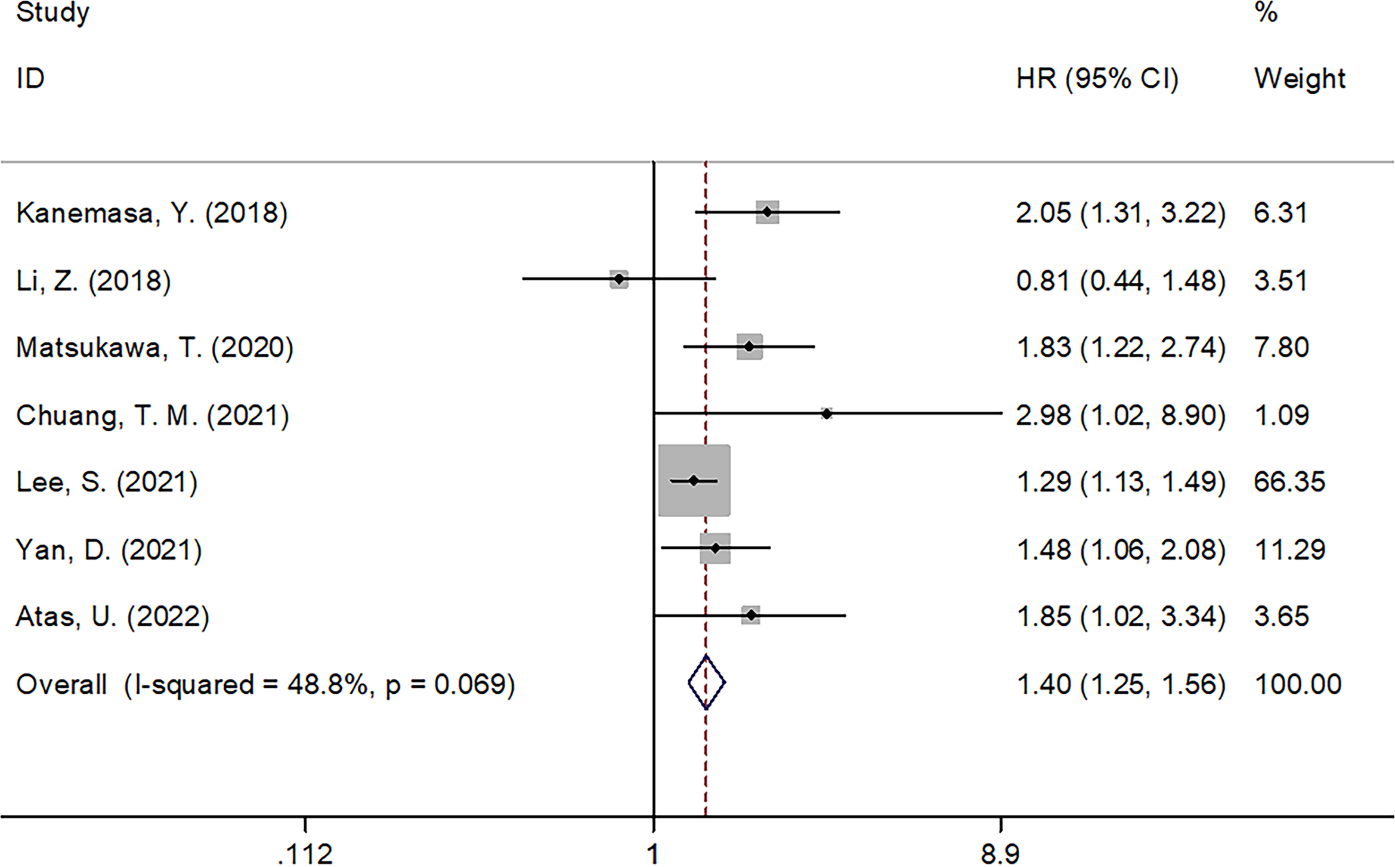
Forest plot of the association of GNRI with OS in patients with DLBCL.

**Figure 3:**
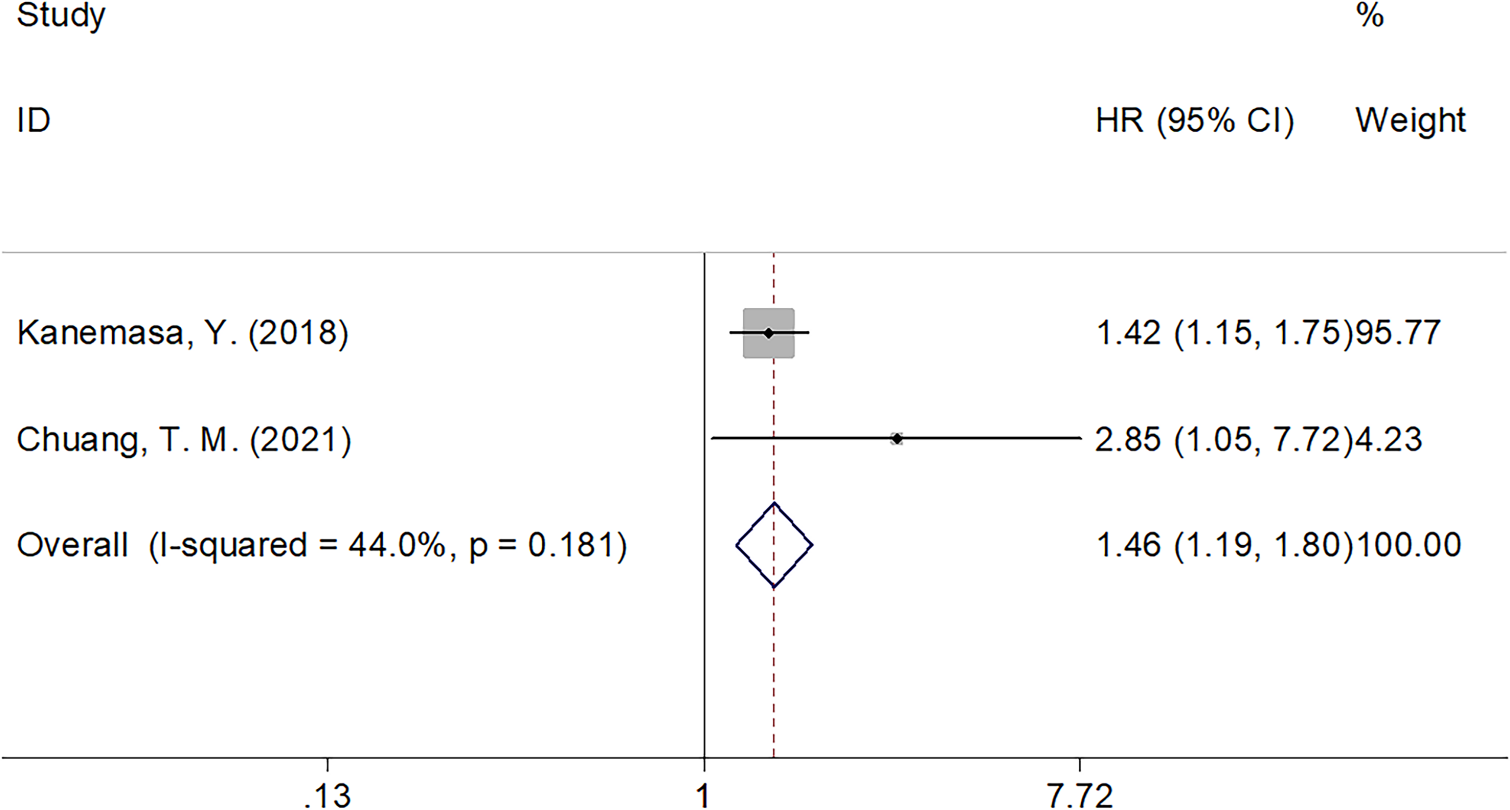
Forest plot of the association of GNRI with PFS in patients with DLBCL.

Seven articles with 2,353 patients (14–20) reported GNRI values for predicting OS in DLBCL. No obvious heterogeneity (I^2 =^ 48.8%, p=0.069) was detected; therefore, we selected a fixed-effects model. According to **Table 2** and **Figure 2**, the combined results were: HR=1.40, 95% CI=1.25–1.56, p<0.001, demonstrating that low GNRI was markedly associated with poor OS in DLBCL. Subgroup analysis by various factors was conducted (**Table 2**), which showed that the reduced GNRI significantly predicted poor OS, regardless of the study center, sample size, treatment, or survival analysis type. Furthermore, a lower GNRI markedly predicted poor OS when using a cut-off value of <98 when the patients’ median age was ≥60 years, and cut-off values were determined using the ROC curve (**Table 2**). Two studies involving 681 patients reported an association between the GNRI and PFS in DLBCL (14, 17). Based on the combined data, a lower GNRI significantly predicted dismal PFS in DLBCL cases (HR=1.46, 95% CI=1.19–1.80, p<0.001; **Figure 3** and **Table 2**).

**Table 2:** Subgroup analysis of the prognostic value of GNRI for OS and PFS in patients with DLBCL.

Factors	No. of studies	No. of patients	Effects model	HR (95%CI)	p	Heterogeneity I^2^(%) Ph	
OS							
Total	7	2,353	Fixed	1.40(1.25-1.56)	<0.001	48.8	0.069
Sample size							
<300	4	811	Fixed	1.44(1.11-1.86)	0.005	49.8	0.113
≥300	3	1,542	Random	1.60(1.17-2.19)	0.003	64.7	0.059
Cut-off value							
<98	4	1,747	Random	1.68(1.22-2.31)	0.001	60.3	0.056
≥98	3	606	Random	1.34(0.88-2.02)	0.171	51.8	0.126
Cut-off determination							
ROC curve	6	2,086	Fixed	1.43(1.27-1.60)	<0.001	40.6	0.134
Literature	1	267	–	0.81(0.44-1.48)	0.488	–	–
Study center							
Single center	5	1,287	Fixed	1.57(1.26-1.96)	<0.001	48.5	0.100
Multicenter	2	1,066	Random	1.46(1.06-2.01)	0.022	60.4	0.112
Survival analysis							
Univariate	1	133	–	1.48(1.06-2.07)	0.022	–	–
Multivariate	6	2,220	Random	1.55(1.18-2.04)	0.002	56.8	0.041
Median age (years)							
<60	2	473	Random	1.23(0.54-2.76)	0.623	73.0	0.054
≥60	5	1,880	Fixed	1.41(1.25-1.59)	<0.001	47.7	0.106
Treatment							
R-CHOP	5	1,426	Fixed	1.54(1.24-1.91)	<0.001	42.4	0.139
R-CHOP or R-CHP-COP	2	927	Random	1.55(1.00-2.40)	0.006	72.8	0.055
PFS							
Total	2	681	Fixed	1.46(1.19-1.80)	<0.001	44.0	0.181

ROC, receiver operating characteristic; OS, overall survival; PFS, progression-free survival; GNRI, geriatric nutritional risk index; DLBCL, diffuse large B-cell lymphoma; R-CHOP, rituximab with cyclophosphamide, doxorubicin, vincristine and prednisone; R-THP-COP, rituximab with cyclophosphamide, tetrahydropyranyl adriamycin, vincristine, prednisolone.

### Relationship of GNRI with clinicopathological factors

**Figure 4:**
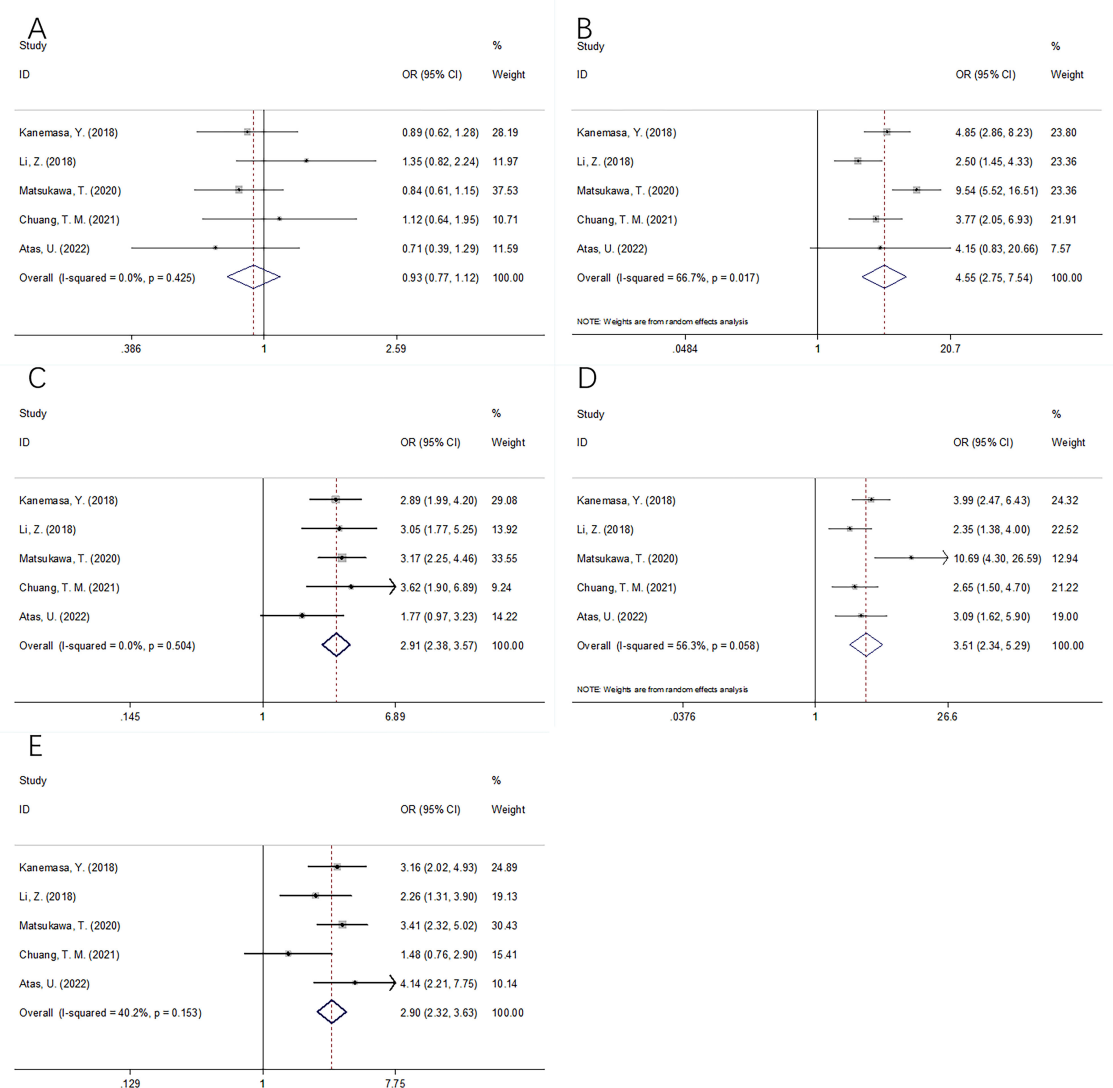
Forest plots of the correlation between GNRI and clinicopathological features in DLBCL patients. **(A)** Gender (male vs female); **(B)** ECOG PS (≥2 vs <2); **(C)** Ann Arbor stage (III-IV vs I-II); **(D)** B symptom (present vs absent); and **(E)** Extranodal disease (yes vs no).

Five studies, involving 1,769 cases, mentioned the correlation between the GNRI and clinicopathological characteristics of DLBCL (14–17, 20). As shown in **Table 3** and **Figure 4**, the combined results revealed a marked relation between the lower GNRI and Eastern Cooperative Oncology Group Performance Status (ECOG-PS) ≥2 (OR=4.55, 95% CI=2.75–7.54, p<0.001), Ann Arbor stage III–IV (OR=2.91, 95% CI=2.38–3.57, p<0.001), B symptoms (OR=3.51, 95% CI=2.34–5.29, p<0.001), and extranodal disease (OR=2.90, 95% CI=2.32–3.63, p<0.001). Nonetheless, the GNRI was not significantly related to sex in DLBCL (OR=0.93, 95% CI=0.77–1.12, p=0.436; **Table 3**; **Figure 4**).

**Table 3:** The association between GNRI and clinicopathological features in patients with DLBCL.

Variables	No. of studies	No. of patients	Effects model	OR (95%CI)	p	Heterogeneity I^2^(%) Ph	
Gender (male vs female)	5	1,769	Fixed	0.93(0.77-1.12)	0.436	0	0.425
ECOG PS (≥2 vs <2)	5	1,769	Random	4.55(2.75-7.54)	<0.001	66.7	0.017
Ann Arbor stage (III-IV vs I-II)	5	1,769	Fixed	2.91(2.38-3.57)	<0.001	0	0.504
B symptom (present vs absent)	5	1,769	Random	3.51(2.34-5.29)	<0.001	56.3	0.058
Extranodal disease (yes vs no)	5	1,769	Fixed	2.90(2.32-3.63)	<0.001	40.2	0.153

GNRI, geriatric nutritional risk index; DLBCL, diffuse large B-cell lymphoma; ECOG PS, eastern cooperative oncology group performance status.

### Publication bias

**Figure 5:**
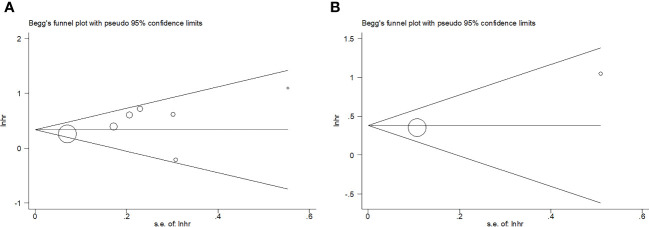
Publication bias. **(A)** OS, Begg’s test, p=0.368; **(B)** PFS, Begg’s test, p=0.317.

We adopted the Begg’s test and funnel plot to examine possible publication bias. As shown in **Figure 5**, symmetry was observed in the funnel plot, and the Begg’s test (p=0.368 and 0.317 for OS and PFS, respectively) revealed no evidence of obvious publication bias.

## Discussion

The role of GNRI in predicting prognosis in patients with DLBCL is controversial based on prior articles. We obtained data from seven articles, comprising 2,353 cases and showed that a lower GNRI markedly predicted worse OS and PFS in DLBCL patients. Based on subgroup analysis, the GNRI reliably predicted OS, especially when the cut-off value was <92. This meta-analysis also revealed that decreased GNRI was significantly associated with clinical factors representing aggressive biological behavior, i.e., ECOG-PS ≥2, Ann Arbor stage III–IV, B symptoms, and extranodal disorder. These factors are well known high-risk factors for disease progression and dismal outcomes in DLBCL cases. Taken together, low GNRI significantly predicted poor OS and PFS in DLBCL patients. To the best of our knowledge, this is the first meta-analysis to analyze the role of GNRI in predicting DLBCL prognosis.

The GNRI is a beneficial tool for assessing nutritional status in clinical practice. There are several cancers for which the GNRI comprises serum albumin levels, BW, and height, which are identified as efficient prognostic factors (13, 23, 24). The correlation mechanism between the GNRI and DLBCL prognosis was interpreted based on components of the GNRI for cancer cases. Approximately 90% of the serum proteins are derived from albumin, which is produced by the liver. Albumin is essential to the human body (25). In addition to reflecting the nutritional status of the human body, serum albumin is a measure of inflammation (26). Albumin has a key effect in maintaining blood colloid osmotic pressure and delivering pharmaceuticals, hormones, cations, and fatty acids (27). Capillary permeability is increased by cancer-related inflammation, which allows serum albumin to escape into the interstitium. Second, >10% BW loss indicates protein-energy malnutrition (28). In comparison with normal-weight cases, underweight DLBCL cases have worse OS and PFS, according to a meta-analysis involving 8,753 participants (29). Patient outcomes have been shown to be significantly affected by weight, as a modifiable factor, and weight management should be aggressive during treatment (30). Finally, low GNRI, possibly caused by weight loss and decreased serum albumin content, is a reasonable and cost-effective prognostic marker for patients with DLBCL.

Notably, previous studies have also explored the prognostic value of several inflammatory parameters, such as the Glasgow prognostic score (GPS) (31), NLR (32), and PLR (33). These studies demonstrated that high GPS and elevated NLR and PLR remained effective prognostic indices for patients with DLBCL (31–33). The GNRI has several advantages and disadvantages compared with GPS, NLR, and PLR. The GNRI is a tool used for nutritional assessment. The nutritional status of patients could be directly reflected by the GNRI, but the GPS, NLR, and PLR have no such function. Second, the GNRI is a novel index that has drawn considerable attention in recent years. The clinical application of the GNRI is much more promising than that of the GPS, NLR, and PLR. However, disadvantages of the GNRI should also be acknowledged. Calculation of the GNRI is more complex than that of the GPS, NLR, and PLR.

We performed a subgroup analysis according to the median age and treatment regimens. As shown in **Table 2**, the results indicated that the GNRI remained a prognostic factor for OS in patients with a median age ≥60 years, and the prognostic role was not influenced by treatment strategies. Therefore, the GNRI could be a reliable prognostic indicator for DLBCL patients aged ≥60 years, whether they received the rituximab, cyclophosphamide, doxorubicin hydrochloride (hydroxydaunorubicin), vincristine sulfate (Oncovin), and prednisone (R-CHOP) or R-CHOP-like regimens. The association between the GNRI and clinicopathological factors was analyzed, and the results are shown in **Figure 4** and **Table 3**. We do not think that these associations are causal because the sample size was relatively sufficient (five studies with 1,769 participants). Moreover, the p-value was <0.001 in these groups, indicating a positive relationship.

Recent meta-analyses have reported that the GNRI significantly predicts cancer prognosis (34–38). Zhang et al. conducted a meta-analysis of 5,593 patients and showed that the GNRI performed well in predicting long-term survival, as well as complications among surgical gastric cancer cases (34). Zhou et al. reported that a low GNRI estimated dismal OS and CSS in esophageal cancer cases in a meta-analysis of 11 studies (39). A recent meta-analysis involving 3,440 participants showed that a lower GNRI before treatment predicted poorer OS and disease-free survival of colorectal cancer cases (40). Another meta-analysis enrolling 6,792 patients indicated that a lower GNRI strongly estimated dismal OS, RFS/PFS, and CSS in urological cancers (38). According to Wang et al., a low GNRI predicted dismal OS, RFS, and CSS in lung cancer cases (41).

Some limitations of the present study should be noted. First, the enrolled articles were retrospective studies, which are not as convincing as randomized controlled trials. Second, all the eligible studies were conducted in Asia. Therefore, the role of GNRI in predicting the prognosis of DLBCL in non-Asian populations should be verified. Third, an optimum cut-off value of the GNRI was not determined in the included studies, which might have caused selection bias. Therefore, large-scale trials in multicenter regions should be conducted for further verification.

## Conclusions

In summary, a low GNRI predicts poorer short- and long-term DLBCL prognosis. A low GNRI was correlated with clinical factors of disease progression in DLBCL.

## Data availability statement

The original contributions presented in the study are included in the article/**Supplementary Material**. Further inquiries can be directed to the corresponding author.

## Author contributions

DC and ZZ designed and conceived the study. DC and ZZ collected the data. DC analyze the data and performed the statistical analysis. ZZ gave the important guidance for statistical analysis and methodology. DC and ZZ provided critical intellectual contributions. DC drafted the manuscript. All authors contributed to the article and approved the submitted version.
