# Supplementary material for: Prognostic and clinicopathological role of geriatric nutritional risk index in patients with diffuse large B-cell lymphoma: A meta-analysis
Source: Front Oncol. 2023 Mar 30;13:1169749. doi: 10.3389/fonc.2023.1169749 (PMC10098099; doi:10.3389/fonc.2023.1169749)
Supplement: Supplementary file 1 [file DataSheet_1.docx]

**Supplementary file 1. The detailed search strategies for each database.**

**1. Search strategies for PubMed:**

Search: (Geriatric Nutritional Risk Index or GNRI) and (diffuse large B-cell lymphoma or lymphoma large B-cell or DLBCL or lymphoma) Sort by: Most Recent

((("geriatric"[All Fields] OR "geriatrics"[MeSH Terms] OR "geriatrics"[All Fields]) AND ("nutrition s"[All Fields] OR "nutritional status"[MeSH Terms] OR ("nutritional"[All Fields] AND "status"[All Fields]) OR "nutritional status"[All Fields] OR "nutrition"[All Fields] OR "nutritional sciences"[MeSH Terms] OR ("nutritional"[All Fields] AND "sciences"[All Fields]) OR "nutritional sciences"[All Fields] OR "nutritional"[All Fields] OR "nutritionals"[All Fields] OR "nutritions"[All Fields] OR "nutritive"[All Fields]) AND ("risk"[MeSH Terms] OR "risk"[All Fields]) AND ("abstracting and indexing"[MeSH Terms] OR ("abstracting"[All Fields] AND "indexing"[All Fields]) OR "abstracting and indexing"[All Fields] OR "index"[All Fields] OR "indexed"[All Fields] OR "indexes"[All Fields] OR "indexing"[All Fields] OR "indexation"[All Fields] OR "indexations"[All Fields] OR "indexe"[All Fields] OR "indexer"[All Fields] OR "indexers"[All Fields] OR "indexs"[All Fields])) OR "GNRI"[All Fields]) AND ("lymphoma, large b cell, diffuse"[MeSH Terms] OR ("lymphoma"[All Fields] AND "large"[All Fields] AND "b cell"[All Fields] AND "diffuse"[All Fields]) OR "diffuse large b-cell lymphoma"[All Fields] OR "diffuse large b cell lymphoma"[All Fields] OR (("lymphoma"[MeSH Terms] OR "lymphoma"[All Fields] OR "lymphomas"[All Fields] OR "lymphoma s"[All Fields]) AND ("large"[All Fields] OR "largely"[All Fields] OR "larges"[All Fields]) AND ("b lymphocytes"[MeSH Terms] OR "b lymphocytes"[All Fields] OR "b cell"[All Fields])) OR ("dlbcl"[All Fields] OR "dlbcls"[All Fields]) OR ("lymphoma"[MeSH Terms] OR "lymphoma"[All Fields] OR "lymphomas"[All Fields] OR "lymphoma s"[All Fields]))

Translations

Geriatric: "geriatric"[All Fields] OR "geriatrics"[MeSH Terms] OR "geriatrics"[All Fields]

Nutritional: "nutrition's"[All Fields] OR "nutritional status"[MeSH Terms] OR ("nutritional"[All Fields] AND "status"[All Fields]) OR "nutritional status"[All Fields] OR "nutrition"[All Fields] OR "nutritional sciences"[MeSH Terms] OR ("nutritional"[All Fields] AND "sciences"[All Fields]) OR "nutritional sciences"[All Fields] OR "nutritional"[All Fields] OR "nutritionals"[All Fields] OR "nutritions"[All Fields] OR "nutritive"[All Fields]

Risk: "risk"[MeSH Terms] OR "risk"[All Fields]

Index: "abstracting and indexing"[MeSH Terms] OR ("abstracting"[All Fields] AND "indexing"[All Fields]) OR "abstracting and indexing"[All Fields] OR "index"[All Fields] OR "indexed"[All Fields] OR "indexes"[All Fields] OR "indexing"[All Fields] OR "indexation"[All Fields] OR "indexations"[All Fields] OR "indexe"[All Fields] OR "indexer"[All Fields] OR "indexers"[All Fields] OR "indexs"[All Fields]

diffuse large B-cell lymphoma: "lymphoma, large b-cell, diffuse"[MeSH Terms] OR ("lymphoma"[All Fields] AND "large"[All Fields] AND "b-cell"[All Fields] AND "diffuse"[All Fields]) OR "diffuse large b-cell lymphoma"[All Fields] OR "diffuse large b cell lymphoma"[All Fields]

lymphoma: "lymphoma"[MeSH Terms] OR "lymphoma"[All Fields] OR "lymphomas"[All Fields] OR "lymphoma's"[All Fields]

large: "large"[All Fields] OR "largely"[All Fields] OR "larges"[All Fields]

B-cell: "b-lymphocytes"[MeSH Terms] OR "b-lymphocytes"[All Fields] OR "b cell"[All Fields]

DLBCL: "dlbcl"[All Fields] OR "dlbcls"[All Fields]

lymphoma: "lymphoma"[MeSH Terms] OR "lymphoma"[All Fields] OR "lymphomas"[All Fields] OR "lymphoma's"[All Fields]

**2. Search strategies for Web of Science:**

ALL=(Geriatric Nutritional Risk Index or GNRI) and (diffuse large B-cell lymphoma or lymphoma large B-cell or DLBCL or lymphoma)

**3. Search strategies for Embase:**

('geriatric nutritional risk index'/exp OR 'geriatric nutritional risk index' OR (('geriatric'/exp OR geriatric) AND nutritional AND ('risk'/exp OR risk) AND ('index'/exp OR index)) OR gnri) AND ('diffuse large b-cell lymphoma'/exp OR 'diffuse large b-cell lymphoma' OR (diffuse AND large AND ('b cell'/exp OR 'b cell') AND ('lymphoma'/exp OR lymphoma)) OR 'lymphoma large b-cell' OR (('lymphoma'/exp OR lymphoma) AND large AND ('b cell'/exp OR 'b cell')) OR 'dlbcl'/exp OR dlbcl OR 'lymphoma'/exp OR lymphoma)

**4.** **Search strategies for Cochrane Library:**

(Geriatric Nutritional Risk Index or GNRI) and (diffuse large B-cell lymphoma or lymphoma large B-cell or DLBCL or lymphoma) in Title Abstract Keyword
